# Macroecological patterns of the phytoplankton production of polyunsaturated aldehydes

**DOI:** 10.1038/s41598-018-29787-8

**Published:** 2018-08-16

**Authors:** Andrés Cózar, Soledad Morillo-García, María J. Ortega, Qian P. Li, Ana Bartual

**Affiliations:** 1 Departamento de Biología, Facultad de Cc. del Mar y Ambientales, Instituto Universitario de Investigación Marina (INMAR), Campus de Excelencia Internacional del Mar CEIMAR, Universidad de Cádiz, E-11510 Puerto Real, Spain; 2 Departamento de Química Orgánica, Facultad de Cc. del Mar y Ambientales, Universidad de Cádiz, E-11510 Puerto Real, Spain; 3 State Key Laboratory of Tropical Oceanography, South China Sea Institute of Oceanology, Chinese Academy of Sciences, 510301 Guangzhou, China

## Abstract

The polyunsaturated aldehydes (PUAs) are bioactive metabolites commonly released by phytoplankton species. Based primarily on laboratory experiments, PUAs have been implicated in deleterious effects on herbivores and competing phytoplankton species or in the regulation of the rates of bacterial organic matter remineralization; however, the role of the PUAs at an ecosystem level is still under discussion. Using data of PUA production in natural phytoplankton assemblages over a wide range of conditions, we analyzed macroecological patterns aiming for a comprehensive environmental contextualization that will further our understanding of the control and ecologic role played by these compounds. PUA composition changed from the predominance of decadienal in oligotrophy, octadienal in eutrophy, and heptadienal at intermediate conditions. The production of PUAs per unit biomass also showed a strong relationship with the trophic status, sharply increasing towards oligotrophic conditions and with small-sized cells reaching the highest production rates. High ratios of dissolved inorganic nitrogen to dissolved inorganic phosphorus also promoted PUA production, albeit to a considerably lesser extent. Although the allelopathic use of PUAs to outcompete other phytoplankton or reduce herbivory may be key in some environments and interactions, the macroecological patterns found here, showing higher production towards the poorest waters and among the small species typically populating these environments, support and link at the large scale the hypotheses of the nutrient-derived stress as driver for the production of PUAs together with the use of these compounds as boosters for the nutrient remineralization.

## Introduction

The polyunsaturated aldehydes (PUAs) are bioactive compounds mostly derived from oxidation of fatty acids released by phytoplankton after cell wounding^1–3^. PUAs can be produced in both marine and freshwater ecosystems, but the capacity to produce PUAs is species and strain specific^4^. Most of the producer species have been identified among the diatoms, although producers may be also found in other phytoplankton groups^5^. PUAs seem to be ubiquitously distributed across the plankton ecosystems^6–8^, and this extensive occurrence suggests a relevant ecological role of the PUAs at the large scale. A rising number of studies are highlighting its relevance as deleterious for the reproduction and development of grazers^9–11^, competing phytoplankton species^12–14^, or as a potential stimulator for the remineralization of the phytoplankton-derived sinking particles^15^.

Our understanding about the PUAs has advanced considerably over the last decade, but is still in its infancy and basic questions remain yet unresolved. The diversity of PUA producers is already wide, but likely many other species are also able to produce the compounds and these kinds of producers will continue to grow as we increase our sampling. PUA concentrations vary greatly from place to place, but the lack of extensive field data analysis prevents a comprehensive understanding about the environmental control of its distribution. These limitations in our knowledge hinder the identification of the main role played by the PUAs in plankton ecosystems. They are suggested to affect various levels in the intricate plankton food webs (i.e. copepods, microzooplankton, recycling bacteria and other phytoplankton species), however the unraveling of the net trophic effect on PUA-producing species is complex^16,17^.

An environmental contextualization of PUA production in nature would constitute an important step towards understanding the control and ecological role played by these bioactive compounds. Interestingly, based on open-ocean samples of large-sized phytoplankton (>10 µm), Bartual *et al*.^6^ found relatively high PUA production in oligotrophic environments. In the present work, we have assembled a dataset of total PUAs in whole phytoplankton communities from systems covering a wide range of environmental conditions in order to characterize macroecological patterns. The approach aims to characterize the shape and boundaries of large-scale biological data in relation to environmental variability. One of the most significant macroecological patterns reported for the phytoplankton concerns the environmental control linked to the resource supply on the community size structure^18^. When resource supply is low, phytoplankton is dominated by small-sized cells, while the fraction of large-sized cells increases as the resources increase, becoming dominant in highly productive ecosystems^18,19^. These contrasting community structures also relate to different modes of ecosystem functioning. Under scarceness of resource, the ecosystem should maximize resource recycling, while new production based on inputs from external sources generally supports highly productive systems with a high abundance of large cells^20^. Based on this robust macroecological pattern, here we analyzed separately the PUA content in the small- and large-size fractions of the phytoplankton community with the objective of determining whether PUA production is also dependent on these different modes of ecosystem functioning. Given that the scant primary production in the systems dominated by small phytoplankton appears sustained by an efficient recycling of resources, we hypothesize that a significant stimulatory effect of the PUAs on the remineralization activity should have led, at the macroscale, to a higher capacity of PUA production in the small-size fraction of the phytoplankton. Moreover, this capacity should be particularly evident in conditions of low resource supply.

## Results and Discussion

**Figure 1:**
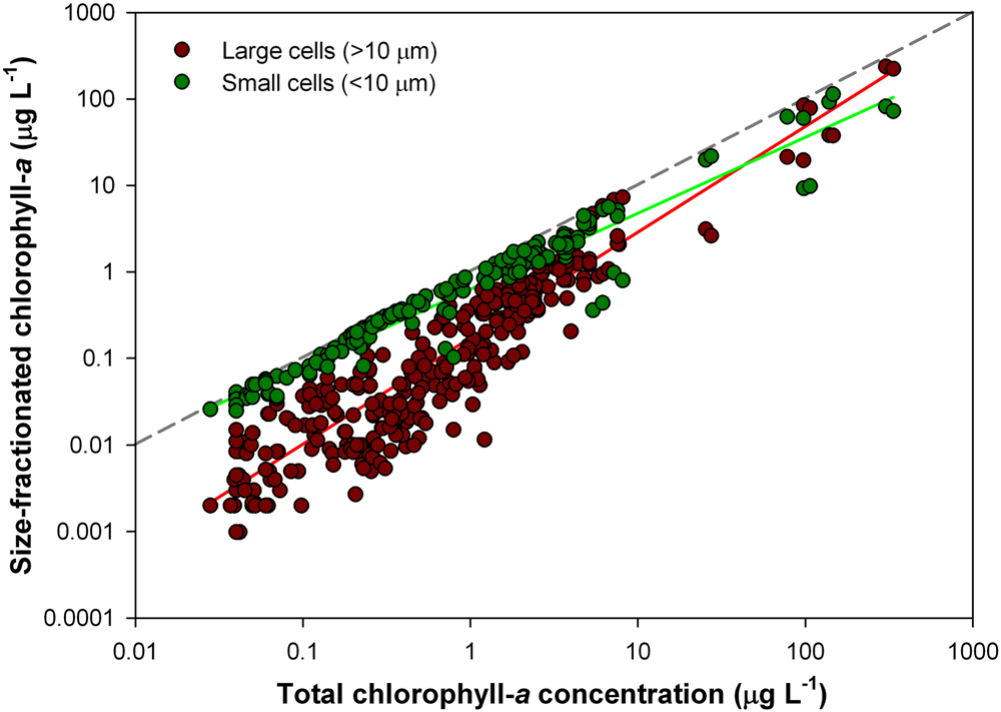
Size-fractionated chlorophyll-*a* (< and >10 μm) in relation to total standing stock of chlorophyll-*a* biomass (*TChl*) in the whole dataset assembled for this study. Red line represents power regression for large-cells data (R = 0.944, p < 0.001) and green line for small-cells data (R = 0.962, p < 0.001). The dashed reference line indicates a 100% contribution to *TChl*.

**Figure 2:**
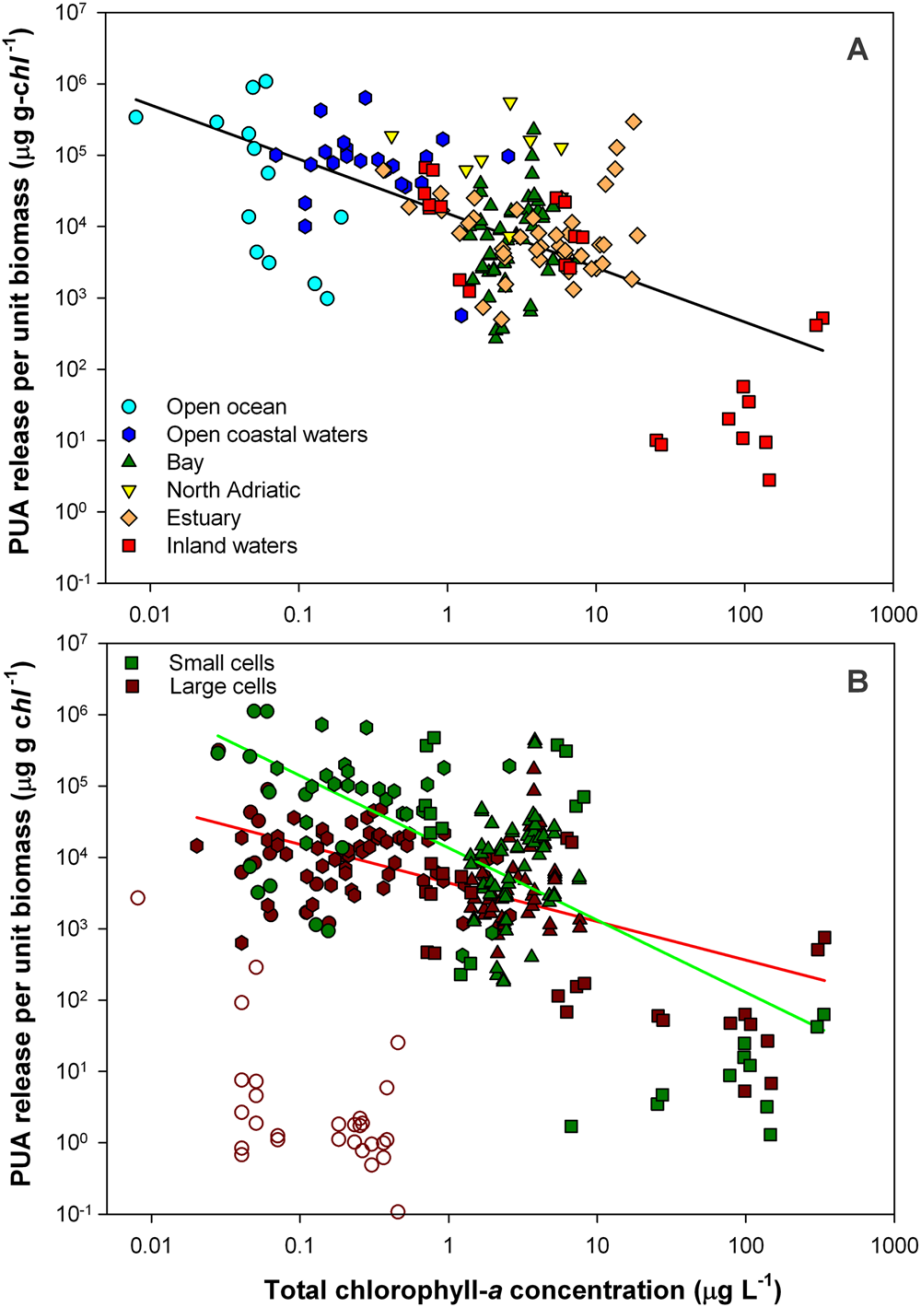
PUA release per unit biomass (μg g-*chl*^−1^) in relation to the total chlorophyll-*a* biomass (*TChl*). Whole phytoplankton assemblage (**A**), and small-size and large-size fractions (**B**). Black line represents power regression for the whole (R = −0.593, p = 0.003), red line for the large-cells data (R = −0.579, p < 0.000) and green line for small-cells data (R = −0.643, p < 0.001). Empty symbols account for samples belonging to the Atlantic transect from Dominican Republic to NW Spain, which are not included in the regression for large cells (see explanation in the text). Note that even removing these data with low PUA production from the large-size dataset, the regression line for small cells was above that for large cells.

**Figure 3:**
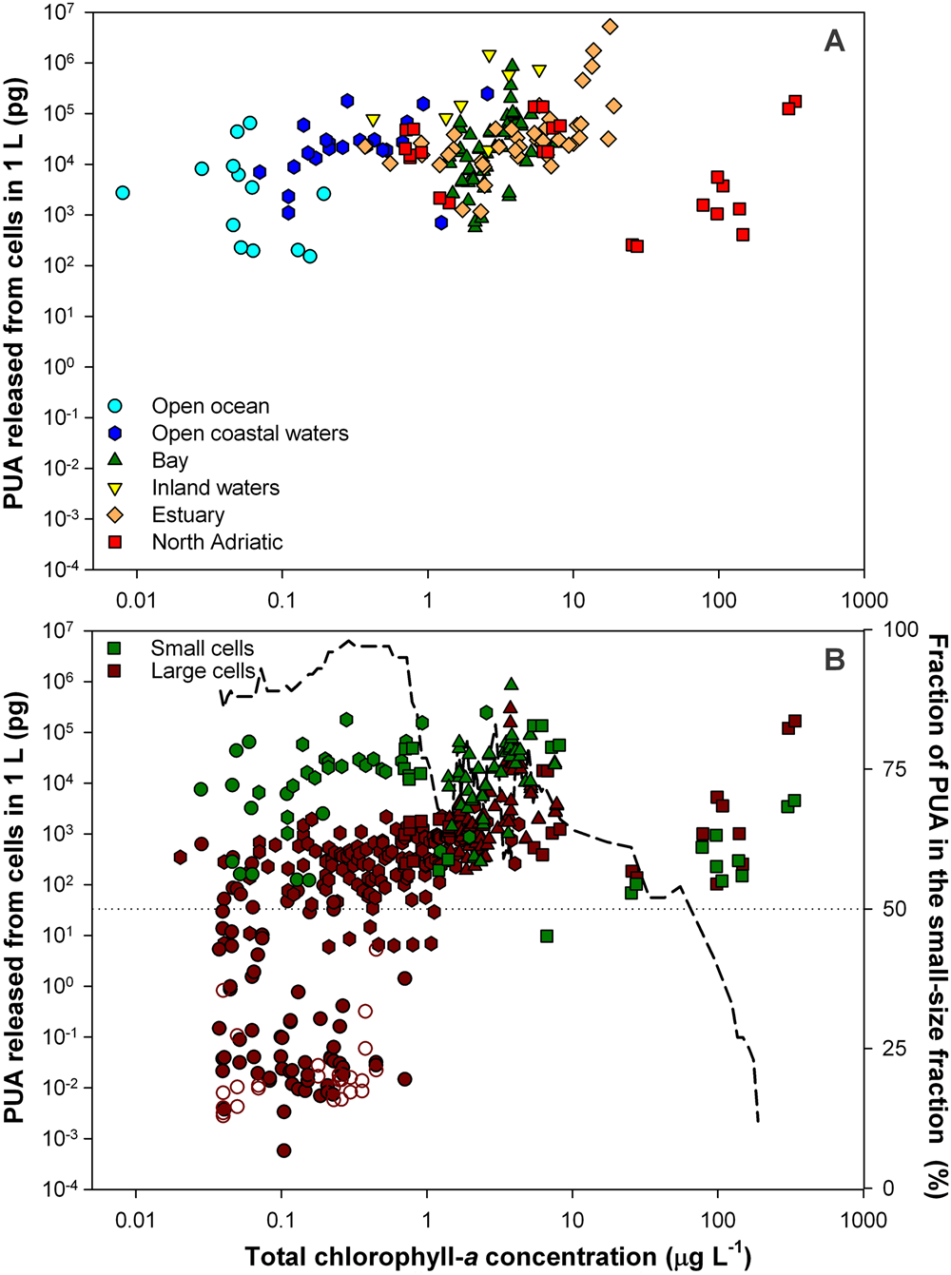
Total PUA released from the cells in 1-L water sample (μg L^−1^) in relation to the total chlorophyll-*a* biomass (*TChl*). Whole phytoplankton assemblage (**A**), and small-size and large-size fractions (**B**). Dashed line in the lower graph shows the fraction of the total PUA released from the small-size fraction (<10 μm), estimated by 11-data moving average.

**Figure 4:**
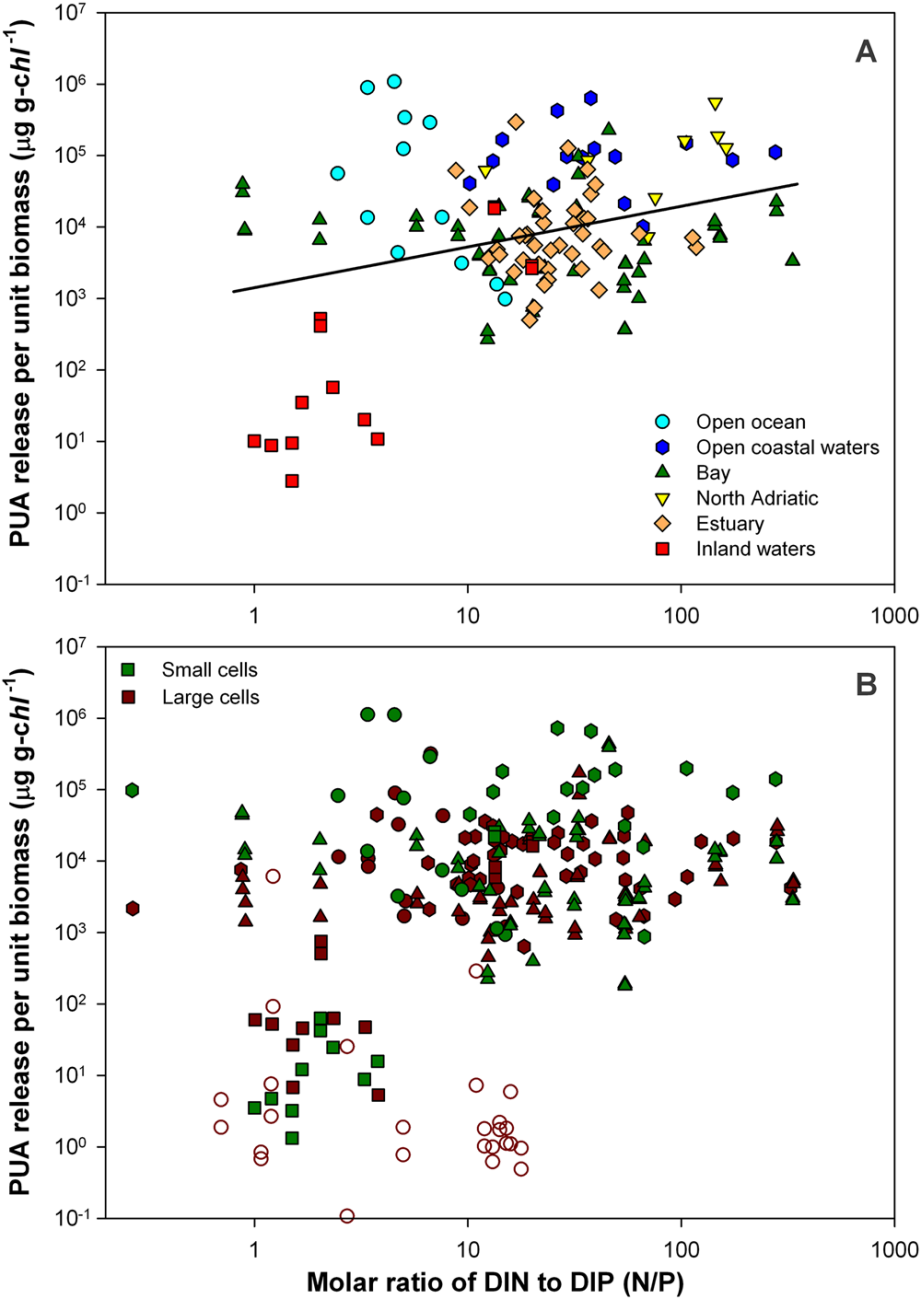
PUA release per unit biomass (μg g-*chl*^−1^) in relation to the molar ratio of DIN to DIP (N/P). Whole phytoplankton assemblage (**A**), and small-size and large-size fractions (**B**). Empty symbols account for samples belonging to the Atlantic transect from Dominican Republic to NW Spain.

The whole dataset assembled here included samples collected from 435 sites, some of them sampled at different times, covering from ultra-oligotrophic ocean waters to hyper-eutrophic lakes (Table 1). As expected, phytoplankton biomass progressively moved from strong dominance of small-sized cells (<10 μm) to a dominance of large-sized cells (>10 μm) along the trophic gradient sampled (Fig. 1). PUAs were quantified as total amount contained in the cells present in one liter of water (μg L^−1^) as well as PUA per biomass unit (μg g-*chl*^−1^), using total chlorophyll-*a* (*TChl*), chlorophyll-*a* in cells <10 μm (*SChl*) or in cells >10 μm (*LChl*) to refer PUAs contained in the whole, small or large phytoplankton respectively. Nevertheless, the environmental contextualization of PUA production was mainly focused on the measurements of PUA per biomass unit (μg g-*chl*^−1^) since the total amount per liter of water is dependent on the changes in biomass concentration, which might also be directly or indirectly related to other environmental variables such as nutrients or temperature. The production of PUAs was analyzed in relation to (i) total chlorophyll-*a* concentration (*TChl*), as indicator of trophic status and, indirectly, resource supply for the biological community, given that the phytoplankton biomass represents the inorganic resource supply captured in recent times by autotrophs as well as the resource available for heterotrophs (Figs 2 and 3); (ii) concentrations of dissolved inorganic nitrogen (DIN) and phosphorous (DIP), as indicative of the nutrients availability at the time of collection (Figs S1 to S2 and S7 to S8); (iii) molar ratio of DIN to DIP (N:P), as indicator of the quality of the nutrients available for the phytoplankton (Figs 4 and S6), (iv) and water temperature (Figs S3 and S9).

**Table 1:** Number and source of PUA data for total phytoplankton (*T-*PUA), small-size fraction (*S-*PUA) and large-size fraction (*L-*PUA) datasets.

Data set	Ecosystem	*S*-PUA (<10 μm)	*L*-PUA (>10 μm)	*T-*PUA	Source
Open Ocean	North and South Atlantic	12 (12)	129 (41)	13	Bartual *et al*.^6^
Open coastal waters	Gulf of Cadiz (eastern North Atlantic) and Strait of Gibraltar	23 (23)	154 (56)	22	this study, Morillo-García *et al*.^26^
Bay	Bay of Cadiz (southwestern Spain)	60 (60)	86 (60)	60	this study
Inland waters	Spanish reservoirs, lakes, and temporary ponds	24 (24)	24 (24)	24	this study
Estuary	Pearl River Estuary and South China Sea	—	—	42	Wu and Li^8^
North Adriatic	Northern Adriatic Sea	—	—	8	Ribalet *et al*.^7^
Total		119 (119)	393 (181)	169	

The number between brackets indicates data number including biomass estimate (*TChl* for *T-*PUA, *SChl* for *S-*PUA, and *LChl* for *T-*PUA). The three datasets only shared a small fraction of samplings, 23% of samples were analyzed for PUAs in both large and small cells and the dataset of *T*-PUAs included a 30% of exclusive samples^7,8^.

**Figure 5:**
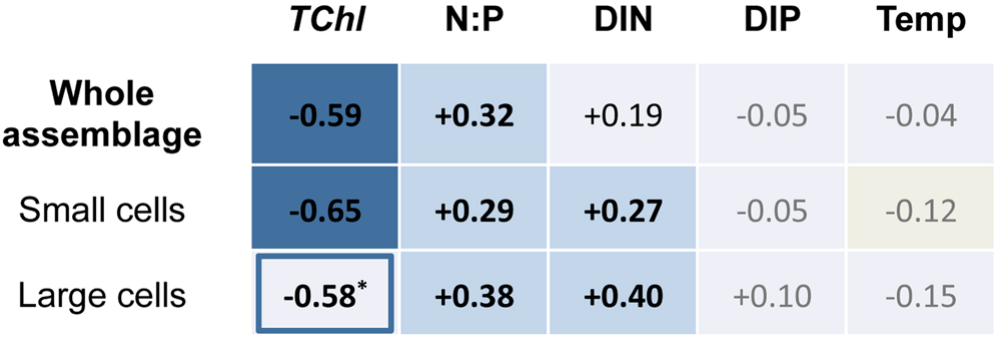
Correlation matrix (R) for power-law relationships between PUAs per unit biomass (μg g-*chl*^−1^) in whole assemblages, small and large cells (< and >10 µm), and total chlorophyll-*a* concentration (*TChl*), concentrations of dissolved inorganic nitrogen (DIN) and phosphorous (DIP), molar ratio of DIN to DIP (N:P), and water temperature (Temp). Blue colour code relates to R values, >0.50 (dark blue), from 0.50 to 0.25 (medium blue), <0.25 (light blue). Significant correlations for p < 0.01 and <0.05 are shown in bold and plain black numbers respectively, while non-significant correlations are in grey numbers. The correlation coefficient shown for PUAs and *TChl* in the large-size fraction excluded samples from the Atlantic transect between Dominican Republic and NW Spain. Including these data (empty symbols in figures), the correlation was not significant, with R decreasing to 0.02.

The most consistent macroecological relationship was found between PUA per unit biomass and trophic status, measured as total chlorophyll-*a* concentration (Figs 5 and S5). PUA production sharply increased towards oligotrophic conditions (Fig. 2). This power-law relationship was apparent in the dataset of whole assemblages as well as in the data for small cells. The replication of the pattern in the small-size fraction is explained by the fact that the smallest cells clearly reached the highest production rates per unit biomass (regression lines in Fig. 2B) and dominated the total PUA production in oligotrophy (Fig. 3B).

The power-law correlation between large-phytoplankton PUAs and *TChl* was made conditional on the removal of the data collected along the Atlantic transect from Dominican Republic to NW Spain (empty symbols in all figures), which showed virtually no production of PUAs. This survey was the only spring oceanic sampling and the northernmost of the transoceanic transects included in the present study^6^. Nevertheless, analyzing a more extensive dataset based on units of PUAs per liter (without requiring biomass estimates for large phytoplankton), we confirm that large-size assemblages with almost no PUA production occurred also in other oligotrophic samples (Figs 3B and S6 to S9). In oligotrophy, PUA in large cells showed a bimodal distribution, with the samples aggregated at significantly high or low PUA production (Fig. S10), indicating that producer species larger than 10 μm were virtually absent from a fraction of the oligotrophic assemblages. Based on the present data collection, the percentage of large-cell assemblages in oligotrophic waters (*TChl* < 1 g L^−1^) showing low PUA production was around one third of the total, ranging from 27% to 38% depending on whether we use PUA per unit biomass or total PUA dataset, respectively.

We hypothesized that the withdrawal of some large-phytoplankton assemblages from the power-law relationship in the poorest waters is due to the general loss of large cells occurring towards the most oligotrophic conditions. In the ocean, there is a sharp reduction in the contribution of large species to total phytoplankton biomass towards oligotrophic conditions^17,18^, as found in our data (Fig. 1). A parallel loss of large PUA-producer species in oligotrophy, preventing their capture in some of the samples of large cells, might explain why some large-phytoplankton assemblages diverged from the general relationship in the poorest waters. In contrast, none of the small-phytoplankton samples diverged from the power-law relationship, suggesting steadier PUA production in this size fraction.

The absolute concentration of PUAs (in μg L^−1^) showed no clear dependence on trophic status (Fig. 3A) because of the trade-off between phytoplankton biomass and production per unit biomass. Nevertheless, the samples with the highest amounts of PUAs were generally found at intermediate interval of the *TChl* gradient, likely associated to a particular growth of highly PUA-productive species. The total amount of PUAs per liter was dominated by the small-size fraction along most of the trophic gradient (Fig. 3B). Only in the most eutrophic sites (*TChl* > 10 µg L^−1^), with majority of large cells and reduced capacity to release PUAs, the contribution of the PUAs derived from large cells overcame those from small cells.

A statistically significant correlation was also found between PUA per unit biomass and N:P ratio, as indicator of the quality of the dissolved inorganic nutrients available at the time of collection (Figs 4 and 5). Hence, the coefficient of multiple correlation (R) between PUA production, *TChl*, and N:P for whole assemblages slightly increased to 0.64 (log PUA (μg g-*chl*^−1^) = 3.64–0.76 · log (*TChl*, g m^−3^) + 0.37 · log (N:P), p < 0.001), compared to the coefficient of correlation between PUA production and *TChl* (R = −0.59). However, the effect of N:P on PUA production was secondary in relation to that of *TChl*. Using the previous equation and the ranges of N:P and *TChl* in our dataset, N:P variability induced a change of one order of magnitude in PUA production, while the effect of the resource supply led to a variation of four orders of magnitude (Fig. S4).

The macroecological pattern found in the present work reveals a higher capacity of PUA production in the most nutrient-impoverished waters and among the small-sized species populating these environments (Fig. 2). Given that it may be expected that communities with low resource supply deal with higher stress level, this pattern matches with the hypothesis that the physiological stress derived from nutrient deficiency triggers the production of PUAs^21^. Likewise, the present findings point to the stimulatory effect on nutrient remineralization proposed by Edwards and co-workers^15^ as possible role of these compounds at the large scale, since the control of the recycling efficiency make particular sense in the poorest waters and among the small species typically adapted to these environments. The proposal by Edwards was based on the response by natural microbial assemblages associated with sinking particles to amendments of varying concentrations of PUAs. Phytoplankton-derived sinking particles emerged as hot spots for PUA production that reach concentrations within the stimulatory range for the particle-associated bacteria^15^. Therefore, the PUA release rate per biomass (Fig. 2), rather than average concentrations into a seawater volume (Fig. 3), takes on special relevance on this context.

**Figure 6:**
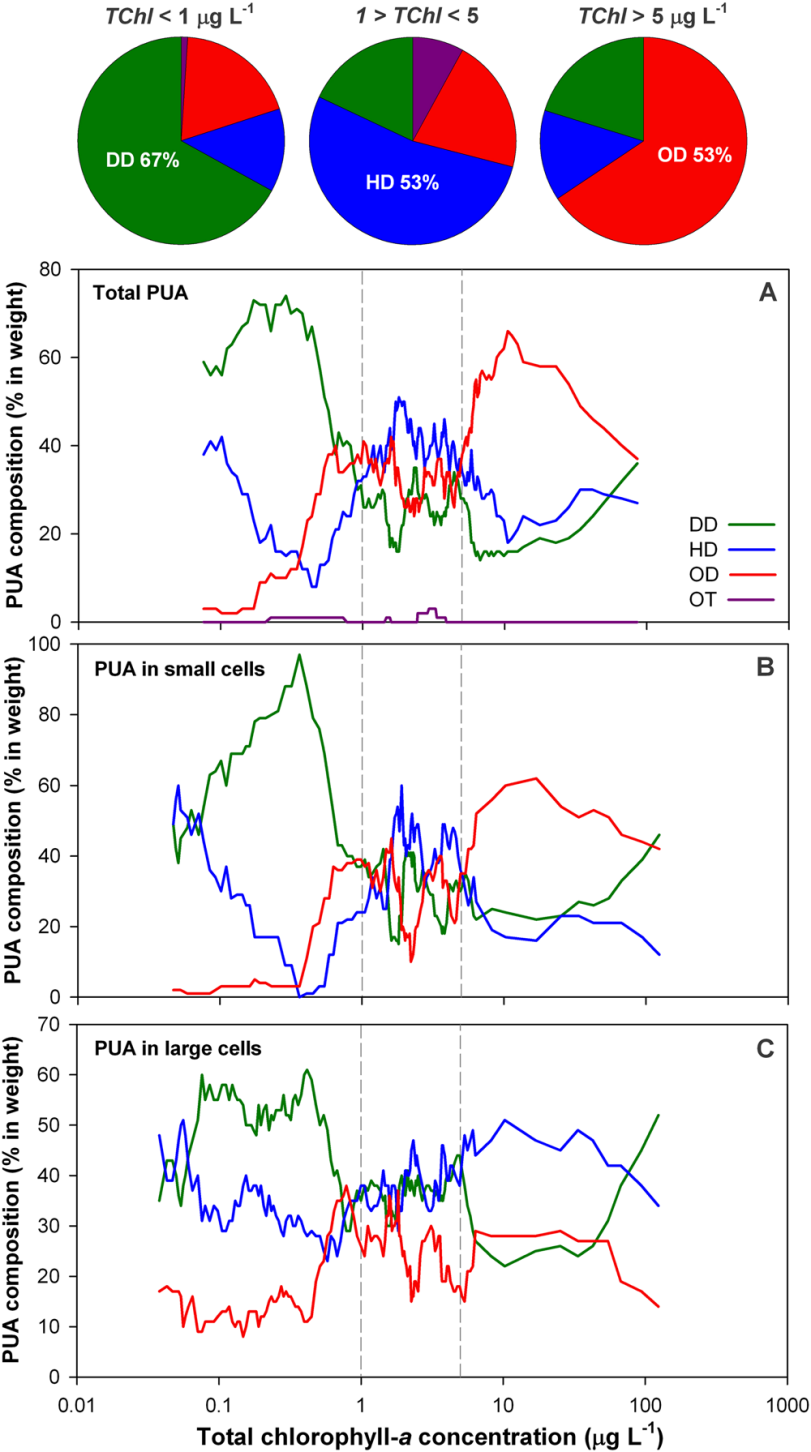
Aldehyde composition of the PUAs released from the whole phytoplankton assemblage (**A**), the small-size fraction (**B**) and the large-size fraction (**C**). Lines correspond to the 11-data moving average for the percentage composition of aldehydes by weight in the individual samples. Vertical reference lines indicate 1 and 5 μg *TChl* L^−1^, and the upper pie charts show the PUA composition in the whole phytoplankton (**A**) for the three trophic intervals delimited by these vertical reference lines. The charts resulted from the addition of amounts of aldehyde measured in all samples within these trophic intervals.

The composition of the PUA pool showed a common pattern in relation to the trophic status in the three datasets, total phytoplankton, small-size fraction and large-size fraction (Fig. 6). We found predominance of decadienal (DD) in oligotrophy (*TChl* < 1 µg L^−1^) and octadienal (OD) in eutrophy (*TChl* > 5.0 µg L^−1^). The highest diversity of aldehydes together with a predominance of heptadienal (HD) was found at intermediate trophic conditions, matching with the highest expected diversity in the phytoplankton^18^. DD is thus the aldehyde mainly associated to the highest PUA production rates, in agreement with the dominant aldehyde found in phytoplankton-derived sinking particles sampled in the North Atlantic^15^.

The observation of a similar change in the aldehyde composition in both size fractions suggests an environmental control rather than a control related to compositional changes in the phytoplankton. The environmental control might involve effects of the resource availability on the aldehyde synthesis or even the possible use of different aldehydes for different purposes. Here, we propose the stimulation of the remineralization in oligotrophy as a major role of the PUAs in nature, however its use as toxic against grazers^22^ and competing species^13^ cannot be discarded since the relevance of the recycling decreases towards eutrophic conditions. On the other hand, DD (prevailing in oligotrophy) has been identified as the most harmful aldehyde in some experiments^23,24^. However, the toxicity experiments have still explored a limited number of species, and understanding the reasons of the intriguing change in composition of PUAs across the trophic gradient warrants specific research attention.

The replication of the compositional pattern of PUAs in the large-size fraction again depended on the removal of the oligotrophic samples collected across the Atlantic transect from Dominican Republic to NW Spain (empty symbols in figures), due to these low-PUA samples showed no production of decadienal (0% DD, 10% OD, 90% HD) while this aldehyde was dominant in the high-PUA samples (PUA > 100 μg g-*chl*^−1^) in oligotrophy (54% DD, 14% OD, 32% HD). These differences were reinforced by examining the larger dataset of absolute PUA concentrations (μg L^−1^) released by large cells in oligotrophy (*TChl* < 1 µg L^−1^). Using 1 pg L^−1^ as threshold between low and high concentrations, DD moved from being the minority aldehyde in the low-PUA samples (13% DD, 33% OD, 54% HD) to be the majority aldehyde in the high-PUA samples (48% DD, 20% OD, 32% HD). Faced with results, we conclude that the contrasting PUA release from the large-size fraction in oligotrophy was largely explained by the lack of large DD-producers.

**Figure 7:**
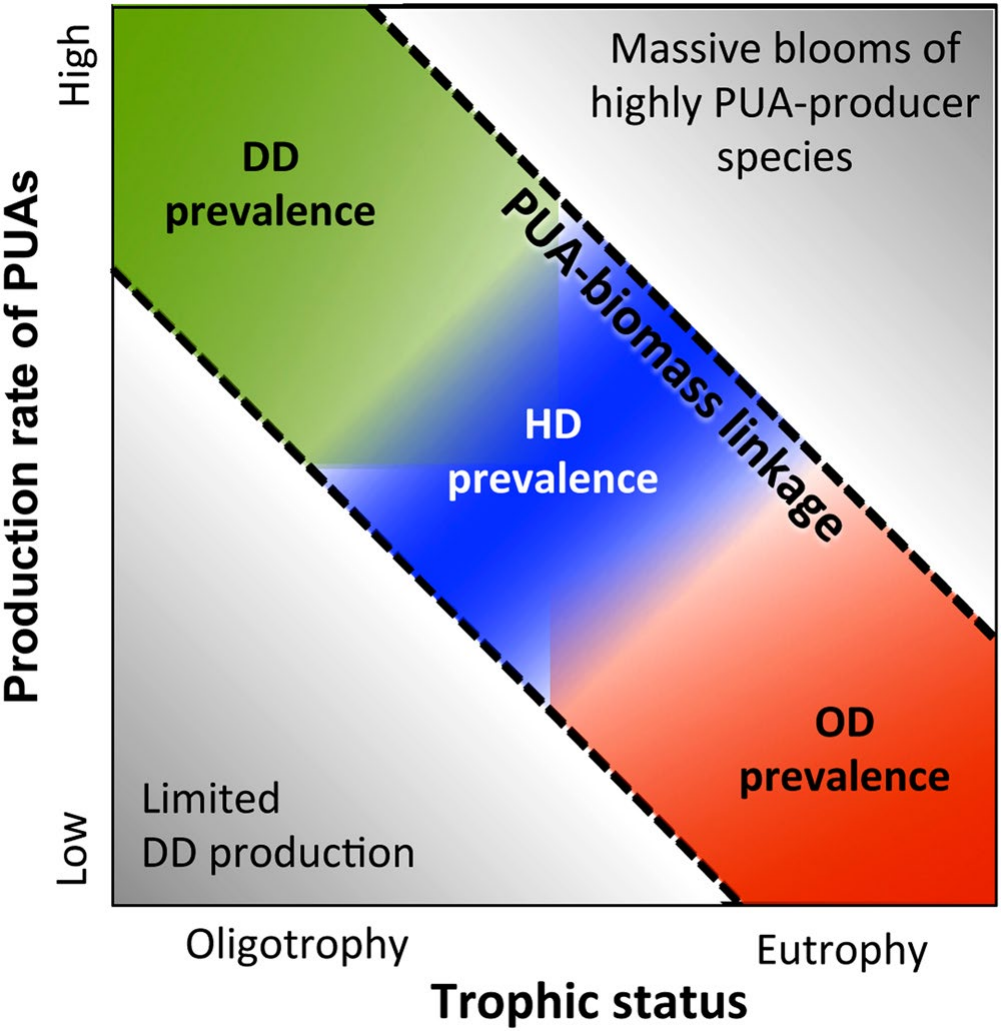
Conceptual diagram describing the macroecological pattern of the PUA production rate per unit biomass in relation to trophic status. Based on Fig. 6, the dominant aldehyde for each trophic interval is shown as DD (decadienal), HD (heptadienal) and OD (octadienal). Figure 2A shows the regression line between PUAs and total chlorophyll-a (*TChl*), while Fig. S4 shows the multiple regression model for PUAs, *TChl* and N:P.

The present work unveils the manner in which the production of PUAs is related to the environmental variability at the macroscale (Fig. 7). Resource supply arises as a main environmental controlling factor. PUA release per unit of phytoplankton biomass, estimated as chlorophyll-*a*, sharply increased towards oligotrophic conditions, likely triggered by nutrient-derived stress^13^. It is known that chlorophyll-*a* is a rough indicator of phytoplankton biomass because the ratio of carbon to chlorophyll-*a* varies across the trophic gradient. However, since this ratio decreases towards oligotrophic conditions^25^, using total carbon instead of chlorophyll-*a* to estimate phytoplankton biomass would make the increase of PUA per unit biomass towards oligotrophy even sharper. In addition, small cells (<10 µm) typically adapted to nutrient-poor environments revealed greater capacity to produce PUAs than large cells. Therefore, the potential usage of PUAs as boosters for nutrient remineralization^15^ takes on a special relevance. However, there are still very few studies supporting the role of PUAs in the bacterial metabolic activity, a hypothesis deserving further research in the light of the patterns found in our data. The highest production rates of PUAs were associated to decadienal (DD). The loss of DD-producers in the large-size fraction resulted in an important reduction in PUA release capacity and the divergence of this specific size fraction from the general linkage between PUAs and trophic status (Fig. 2B). These outliers would be placed in the lower left quadrant of Fig. 7 (low PUA release, low resource supply), however we did not find such outliers by accounting for whole assemblages because of small-size fraction was strongly dominant in biomass in oligotrophy and showed the highest production rates. Our datasets had no samples in the upper right quadrant either (high PUA release, high resource supply). Intense blooms of highly-producer species should theoretically be placed in this quadrant. Indeed, we confirm this possibility by inducing intense growth in laboratory cultures of a PUA-producer species (*Thalasiossira rotula*; *TChl* from 180 to 220 µg L^−1^, PUA from 10^6^ to 10^7^ µg g-*chl*^−1^; unpublished data). The question as to whether there may be cases of natural assemblages falling into the lower left and upper right quadrants of the diagram and the causes undoubtedly requires further sampling, albeit these cases seem to be uncommon in nature according to the available data. We expect that the present environmental contextualization of the PUA production and composition across plankton ecosystems will provide a useful background for further taxonomic analyses, experiment design and future field surveys.

## Methods

A total of 435 sites were sampled in eighteen surveys carried out from October 2008 to June 2015. Sampling sites were placed across a range of freshwater and marine aquatic ecosystems, including offshore and coastal waters, bays, estuaries, lakes, reservoirs and ponds (Table 1). A fraction of the data (36%) was previously discussed in partial studies by us^6,8,26^. In addition, eight average data of total PUAs in phytoplankton assemblages from the Northern Adriatic Sea, reported by Ribalet and co-workers^7^, were included in the dataset. Therefore, the present dataset accounts for virtually all the available field data of particulate PUA up to date.

All samples were collected in the surface layer (<5 m depth) of the water bodies, in agreement with most of previously reported data. Because the surface layer is easier to sample, focus on surface waters allowed for a comparable and more spatially extensive dataset, although we acknowledge that the variability of PUAs throughout the water column remains an important gap in our knowledge^27^. Water temperature was measured during sampling, and supplementary samples were also collected for quantifying concentrations of total chlorophyll-*a* (*TChl*), chlorophyll-*a* in large phytoplankton (>10 μm, *LChl*) and small phytoplankton (<10 μm *SChl*), dissolved inorganic nitrogen (DIN) and dissolved inorganic phosphorus (DIP).

*TChl* was estimated from >0.5 L water samples filtered through Whatman GF/F filters using the fluorimetric method^28,29^. For *LChl*, 2 L of water were filtered through a nylon mesh with a 10-μm nominal pore size. The plankton fraction retained on the mesh was then collected by washing with filtered (Whatman GF/F, 0.7 μm) water, filtered again through a Whatman GF/F filter, immersed in liquid N_2_ and conserved frozen until anlysis. For *SChl*, 1 L of the water passed through the 10-μm nylon mesh was recovered and filtered through a Whatman GF/F filter and conserved frozen until analysis. The chlorophyll content of both *SChl* and *LChla* were determined following the protocol described above for *TChl*.

DIN and DIP were measured using three replicates of filtered water (12 mL, Whatman GF/F filters) for each dissolved inorganic nutrient (nitrite, nitrate, ammonium and phosphate). The samples were stored at −20 °C and analyzed using an autoanalyser (Technicon AA-II -TRACS 800)^30^.

The water volume to analyze PUAs depended on the plankton concentration, ranging from 25 to 400 L, in order to ensure PUA detection in the most oligotrophic waters. The water volume was passed through three consecutive meshes, a first of 200 µm (to remove microzooplankton), a second of 10 µm to collect plankton larger than 10 µm, and finally a nucleopore polycarbonate 0.4 μm pore size filter (GE Poretics^TM^) to retain the small plankton. The phytoplankton samples retained in the 10-µm mesh to estimate PUAs in the large-sized plankton (*L-*PUA) was diluted in 125 ml of filtered seawater each and then passed through a nucleopore polycarbonate filter with a 0.4-µm pore size (GE Poretics^TM^). The filter was transferred to 25 ml glass vial (Teknokroma) and rinsed using 1 ml of a 25 mM *O-*(2,3,4,5,6-Pentafluorobenzyl) hydroxylamine (PFBHA, Fluka, Basel, Switzerland) solution in Tris-HCl 100 mM, pH 7.2 (Trizma, Sigma). This reagent stabilizes aldehydes by derivatization, preventing volatilization and eventual transformation before the analysis by GC-MS. These samples were then stored at −20 °C until further analysis. Simultaneously, the phytoplankton retained in the nucleopore filters were immersed in PFBHA following the above explained protocol, and stored at −20 °C for measuring PUAs produced by the plankton smaller than 10 µm (*S-*PUA). The total PUAs released by the plankton assemblages (*T-*PUA) was estimated by adding the PUAs released by both size fractions, larger and smaller than 10 µm (*L-*PUA + *S-*PUA).

To quantify PUA concentrations, we used the protocol detailed by Morillo-García *et al*.^26^. Briefly, cell suspensions were defrosted and 500 μL of internal standard added (benzaldehyde). Mechanical disruption was accomplished by ultrasound sonication on ice and kept for 1 h at room temperature to ensure derivatization.

For extraction, samples were transferred into a 25 mL glass separating funnel using a ratio 2:1:2 (hexane:methanol:water). The mixture was vortexed for 1–2 min, 3–4 drops of sulphuric acid (95% purity) were added and vortexed again. After separation of the phases, the hexane layer was collected, dried over anhydrous Na_2_SO_4_ and taken to dryness under reduced pressure. The extracts obtained were dissolved in 50 μL of hexane, transferred into 100 μL glass microinsert and subjected to GC-MS analysis.

GC-MS analysis of PUAs were performed using a CE INSTRUMENTS AS 800-GC 8000 TOP/mass spectrometer Finnigan Voyager or a Quattro micro GC (Waters® Micromass® Quattro micro™ GC, Milford, MA, USA) tandem quadrupole instrument, coupled to an Agilent 7890 A gas chromatograph (GC) (Agilent, Santa Clara, CA, USA) equipped with a 30 m DB-5 MS column (0,25 mm internal diameter, 0,25 μm stationary phase thickness, 95% dimethylpolysyloxane 5% diphenylpolysyloxane). The GC oven temperature was programmed as follows: held at 60 °C for 1 min, then increased at 5 °C per minute to 300 °C and finally held at 300 °C for 1 min. The inlet temperature was 250 °C in splitless mode (1 μL). Helium 5.0 was the carrier gas at a constant flow of 1 mL/min. Electron impact (EI) ionization process with an energy of 70 eV in SIR mode was used for analysis following the protocols detailed by Bartual and Ortega^31^ and the data processed using Xcalibur software (version 1.2 from Finningan Corp. 1998–2000) or MassLynx software (version 4.1, Waters, Milford, MA, USA). PUAs were identified by comparing the retention time with standards and by the presence of the molecular ion (*m*/*z* 305 for HD, *m*/*z* 319 for OD and *m*/*z* 347 for DD) and major fragment ions, i.e. *m*/*z* 276 for the PUAs, 271 for the standard and 181 for all. Quantification was based on the ratio between the fragment at *m/z* 276 of the derivatized PUA and the fragment at *m*/*z* 271 of the derivatized internal standard (benzaldehyde). The calibration curves was performed using synthetic standard of derivatized (25 mM PFBHA-solution in TRIS-HCl at pH 7.2) 2*E*, 4*E*-heptadienal (90% Sigma-Aldrich Chemie GmbH, Steinheim, Germany), 2*E*, 4*E*-octadienal (≥96% Sigma-Aldrich Chemie GmbH, Steinheim, Germany), and 2*E*, 4*E*-decadienal (85% Sigma-Aldrich Chemie GmbH, Steinheim, Germany), in ranges 0.5–15 nM, 15–200 nM to 200–7000 nM, to cover the wide range of molarities found in the concentrated extracts of the samples. Calibration curves were freshly prepared and repeated with every group of samples analyzed.

Measurements of PUAs in both size fractions (> and <10 μm) or some of the environmental variables (i.e., water temperature, *TChl*, *SChl, LChl*, DIN, DIP) were not always available because of sampling constraints or simply because this task was not in the original planning. This resulted in differences in the origin and number of data used for analyzing total phytoplankton, small cells and large cells (Table 1).

## Electronic supplementary material

Supplementary Information
