# Supplementary material for: Macroecological patterns of the phytoplankton production of polyunsaturated aldehydes
Source: Sci Rep. 2018 Aug 16;8:12282. doi: 10.1038/s41598-018-29787-8 (PMC6095871; doi:10.1038/s41598-018-29787-8)
Supplement: Supplementary file 1 — Supplementary Information [file 41598_2018_29787_MOESM1_ESM.pdf]

Supplementary Information for:

Macroecological patterns of the phytoplankton production of polyunsaturated aldehydes

Andrés Cózar*, Soledad Morillo-García, María J. Ortega, Qian P. Li, and Ana Bartual

*Corresponding author. E-mail: andres.cozar@uca.es

This PDF file includes:

Fig. S1
Fig. S2
Fig. S3
Fig. S4
Fig. S5
Fig. S6
Fig. S7
Fig. S8
Fig. S9
Fig. S10

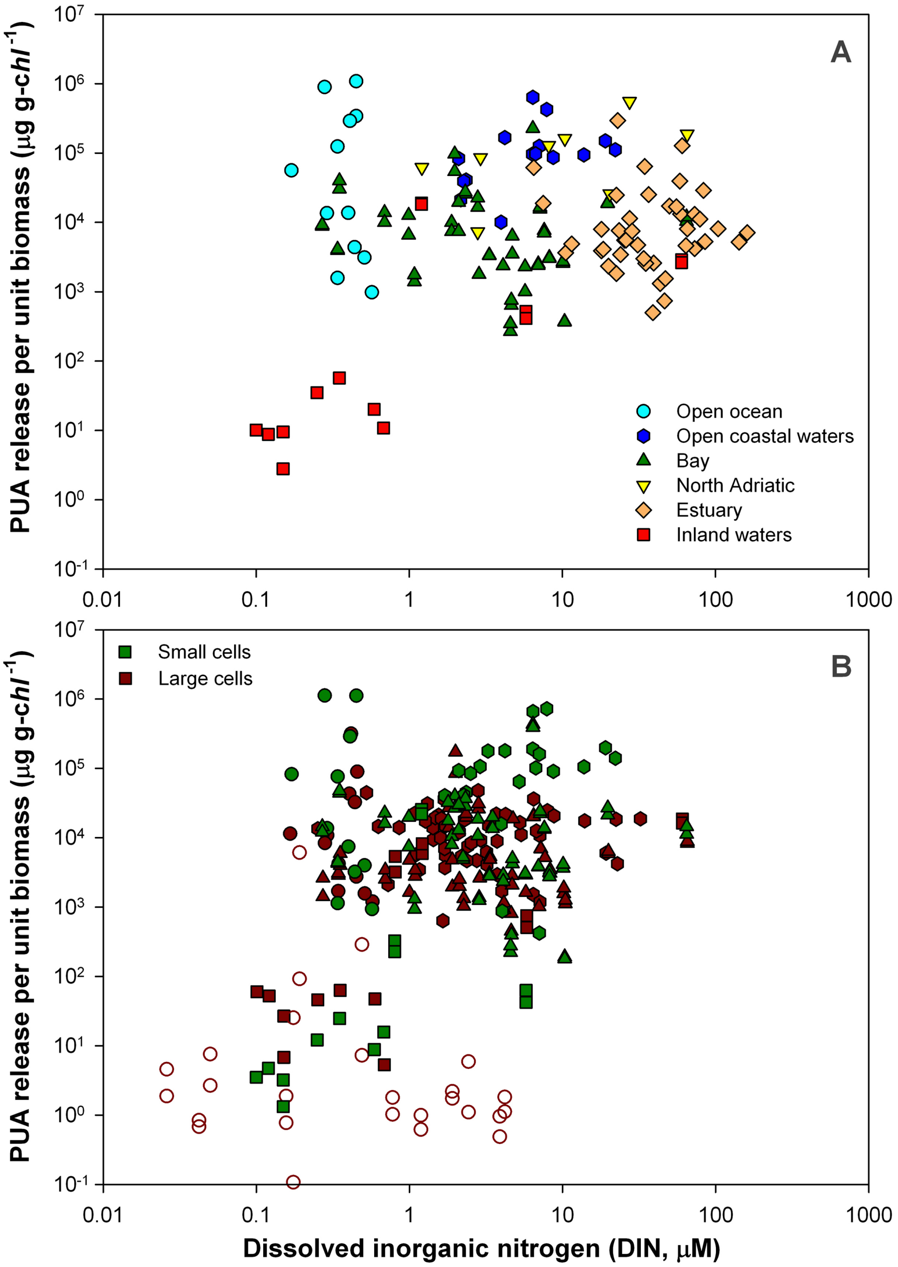

Figure S1: PUA release per unit biomass (µg g-*chl*$^{-1}$) in relation to concentration of dissolved inorganic nitrogen (DIN). Whole phytoplankton assemblage (A), and small-size and large-size fractions (B). Empty symbols account for samples belonging to the Atlantic transect from Dominican Republic to NW Spain.

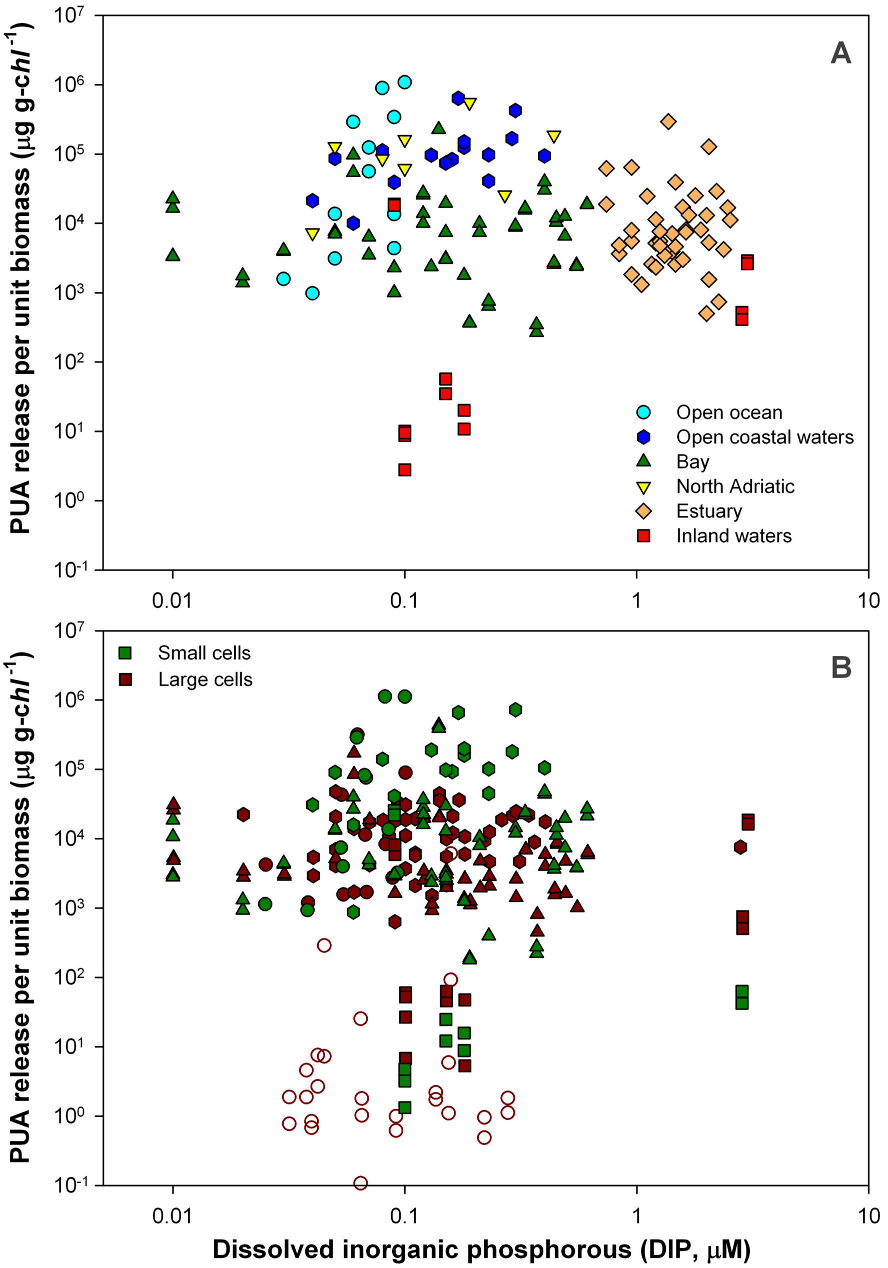

Figure S2: PUA release per unit biomass (µg g-*chl*$^{-1}$) in relation to concentration of dissolved inorganic phosphorous (DIP). Whole phytoplankton assemblage (A), and small-size and large-size fractions (B). Empty symbols account for samples belonging to the Atlantic transect from Dominican Republic to NW Spain.

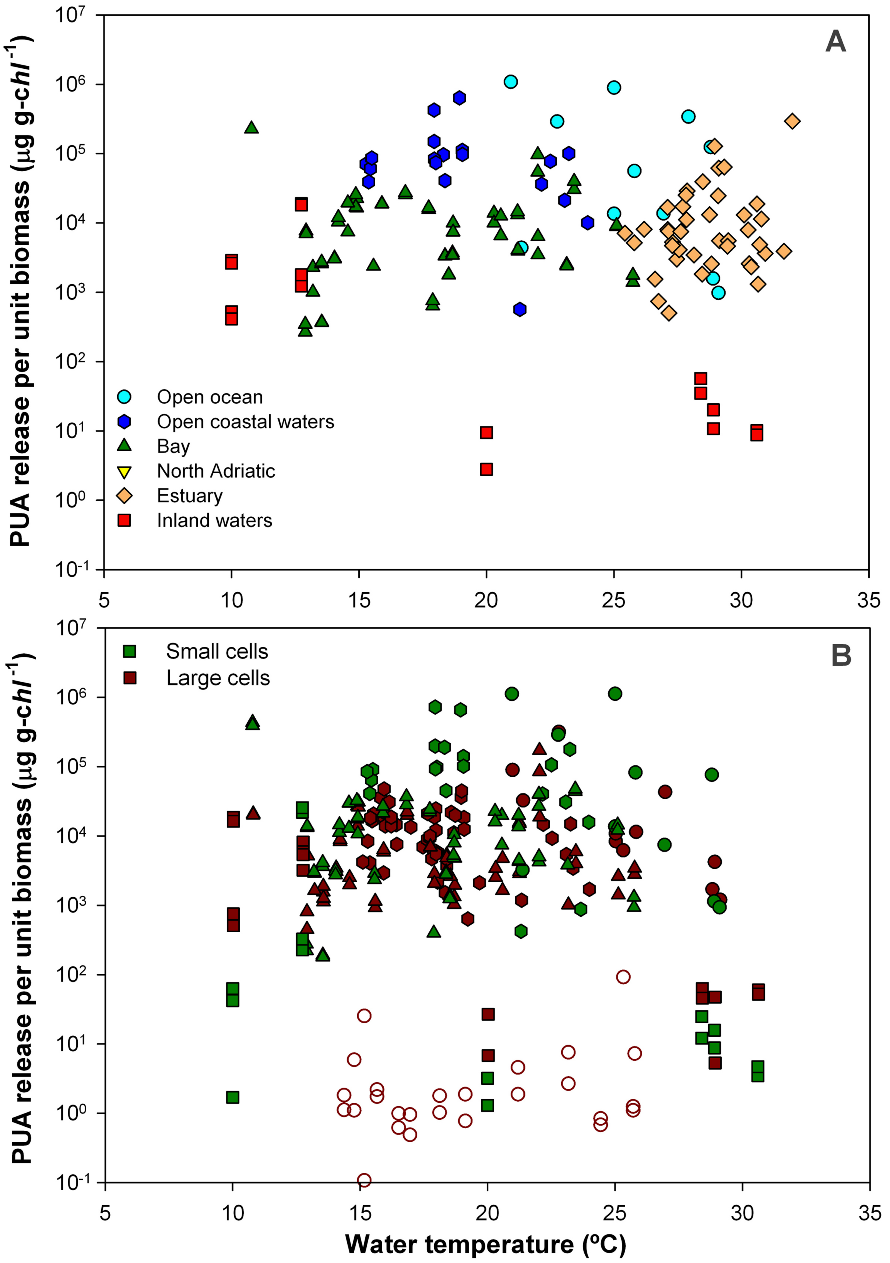

Figure S3: PUA release per unit biomass (µg-PUA g-*chl*$^{-1}$) in relation to water temperature. Whole phytoplankton assemblage (A), and small-size and large-size fractions (B). Empty symbols account for samples belonging to the Atlantic transect from Dominican Republic to NW Spain.

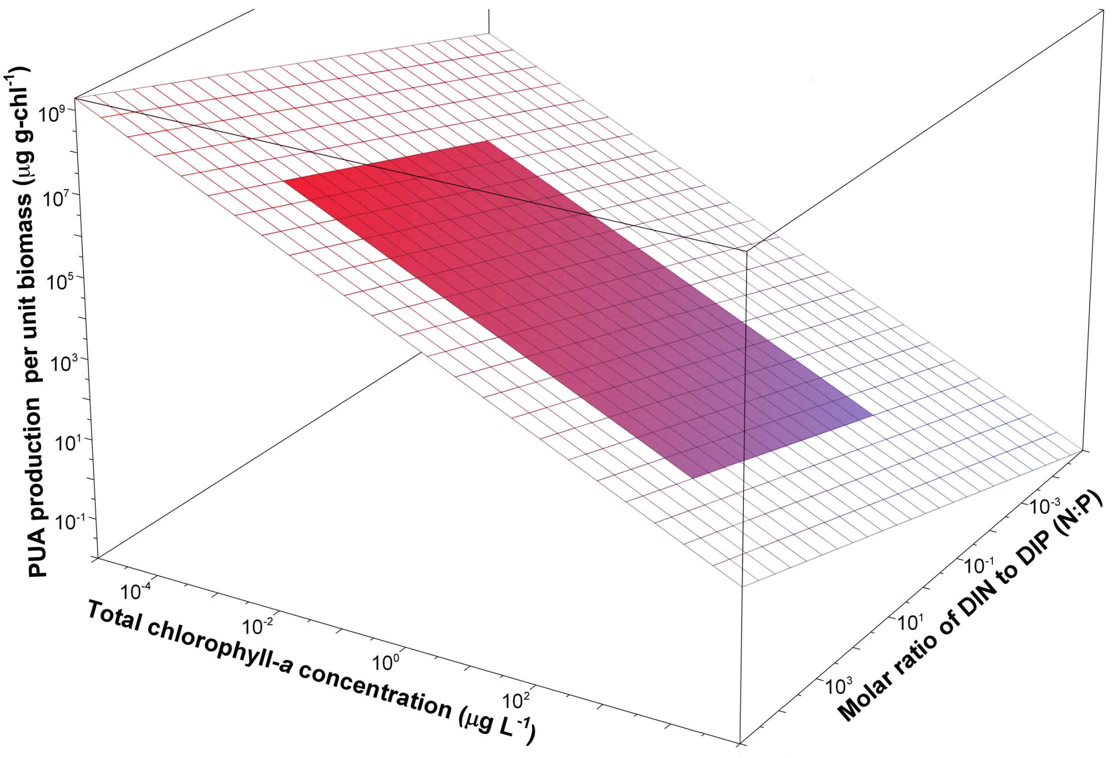

Figure S4: Surface plot describing the relationship between PUAs per unit biomass, total chlorophyll-a (*TChl*) and molar ratio of DIN to DIP (N:P), obtained by multiple regression to data accounting for whole phytoplankton assemblages: log PUA (μg g-chl^{-1}) = 3.64 − 0.76 · log (*TChl*, g m^{-3}) + 0.37 · log (N:P), $R = 0.64$, $p < 0.001$. The coloured surface extends over the ranges of *TChl* and N:P in the present data collection.

	TChl	N:P	DIN	DIP	Temp
**Whole assemblage**	+0.21	**+0.28**	**+0.39**	**+0.24**	-0.06
Small cells	-0.08	**+0.27**	**+0.37**	-0.14	**-0.31**
Large cells	**+0.63**	**+0.24**	**+0.51**	**+0.33**	**-0.47**

Figure S5: Correlation matrix (R) for power-law relationships between total PUA released from the cells in 1-L water sample (µg L^{-1}) for whole assemblages, small and large cells (< and > 10 µm), and total chlorophyll-*a* concentration (*TChl*), concentrations of dissolved inorganic nitrogen (DIN) and phosphorous (DIP), molar ratio of DIN to DIP (N:P), and water temperature (Temp). Blue colour code relates to R values, > 0.50 (dark blue), from 0.50 to 0.25 (medium blue), < 0.25 (light blue). Significant correlations for p < 0.01 and < 0.05 are shown in bold and plain black numbers respectively, while non-significant correlations are in grey numbers.

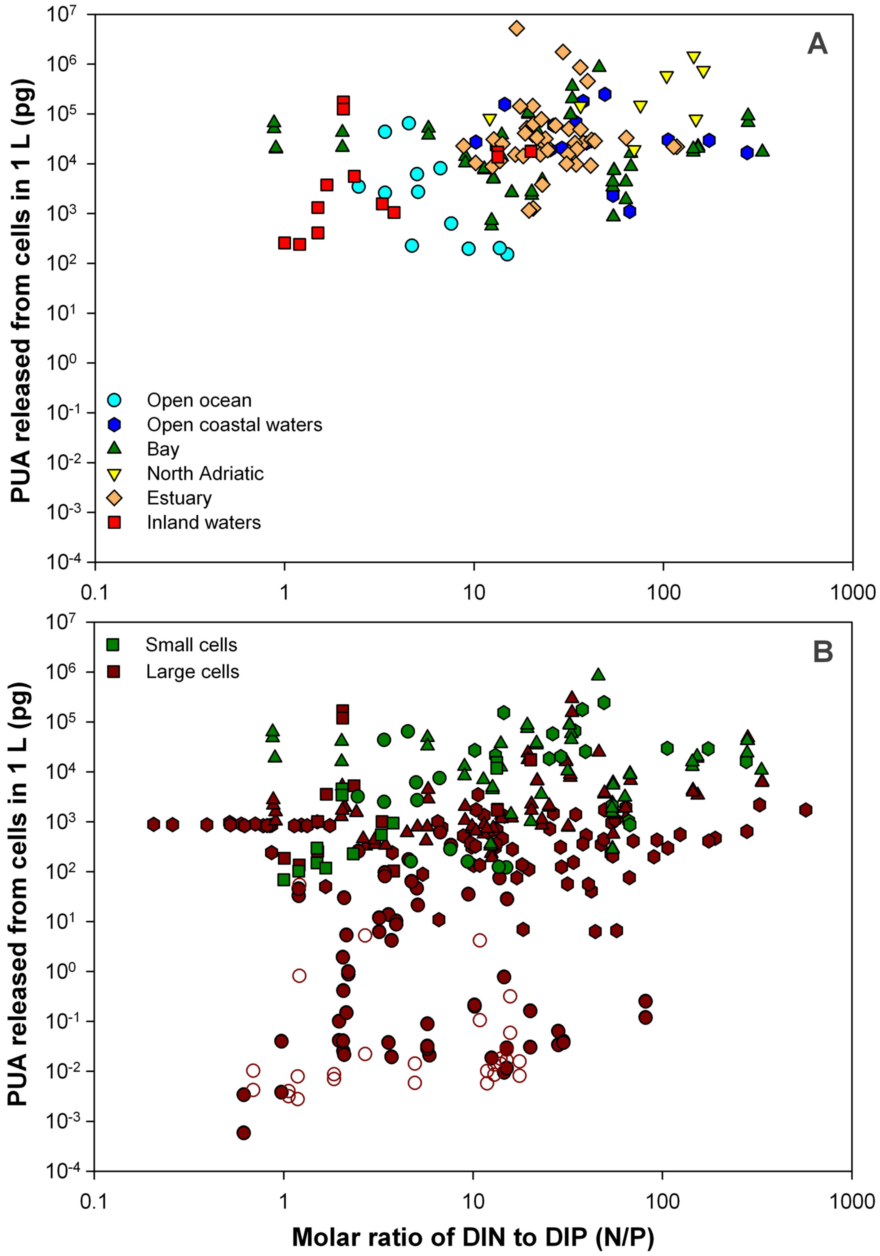

Figure S6: Total PUA released from the cells in 1-L sample (μg L^{-1}) in relation to the molar ratio of DIN to DIP (N/P). Whole phytoplankton assemblage (A), and small-size and large-size fractions (B). Empty symbols account for samples belonging to the Atlantic transect from Dominican Republic to NW Spain.

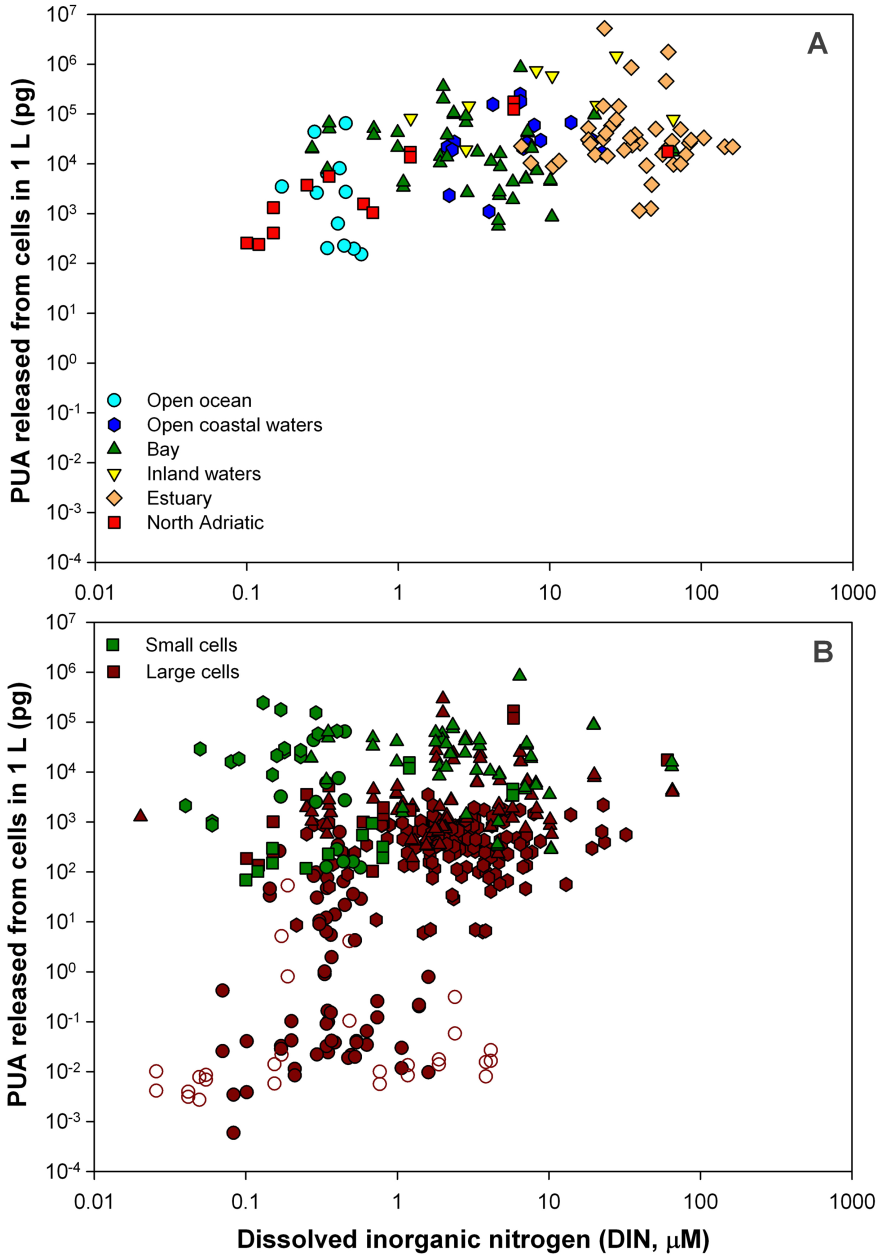

Figure S7: Total PUA released from the cells in 1-L sample (µg L^{-1}) in relation to concentration of dissolved inorganic nitrogen (DIN). Whole phytoplankton assemblage (A), and small-size and large-size fractions (B). Empty symbols account for samples belonging to the Atlantic transect from Dominican Republic to NW Spain.

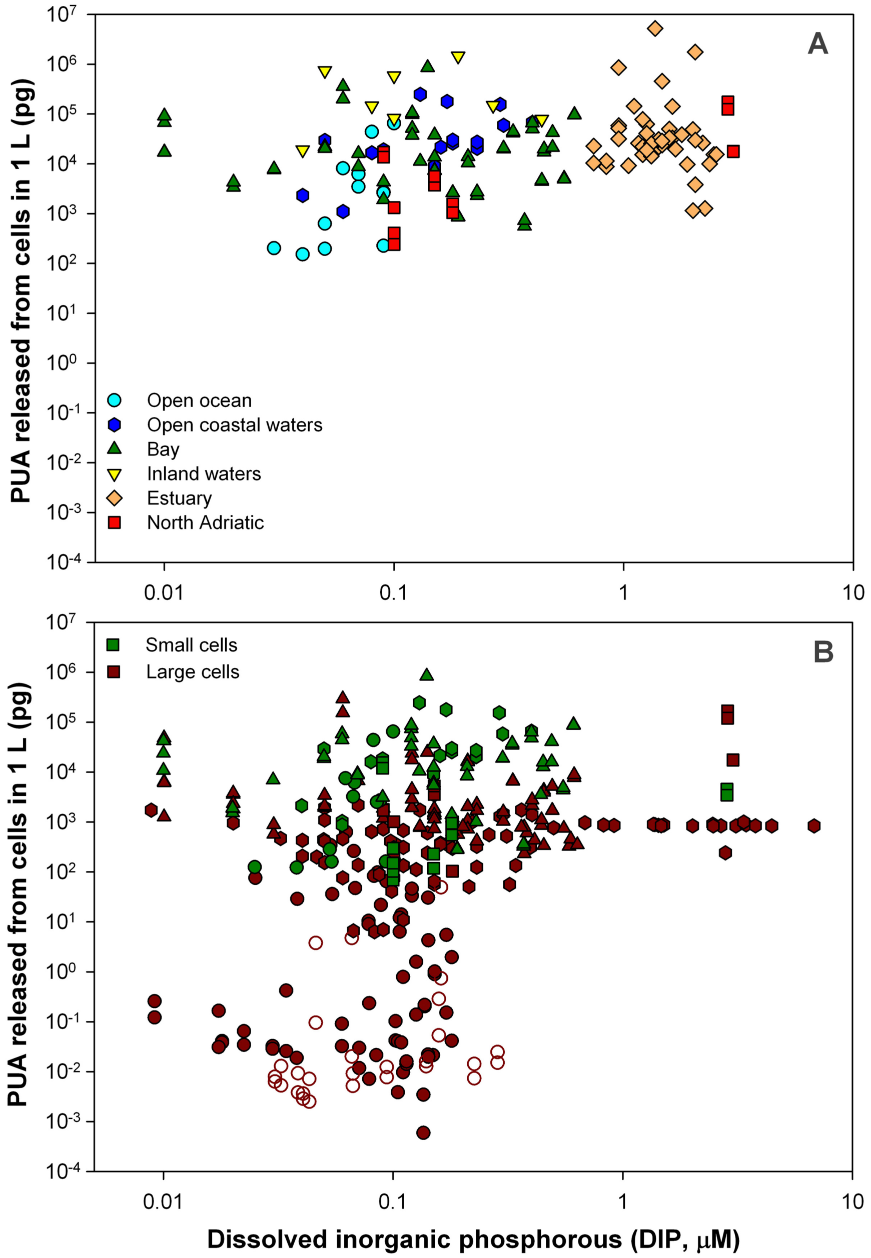

Figure S8: Total PUA released from the cells in 1-L sample (μg L^{-1}) in relation to concentration of dissolved inorganic phosphorous (DIP). Whole phytoplankton assemblage (A), and small-size and large-size fractions (B). Empty symbols account for samples belonging to the Atlantic transect from Dominican Republic to NW Spain.

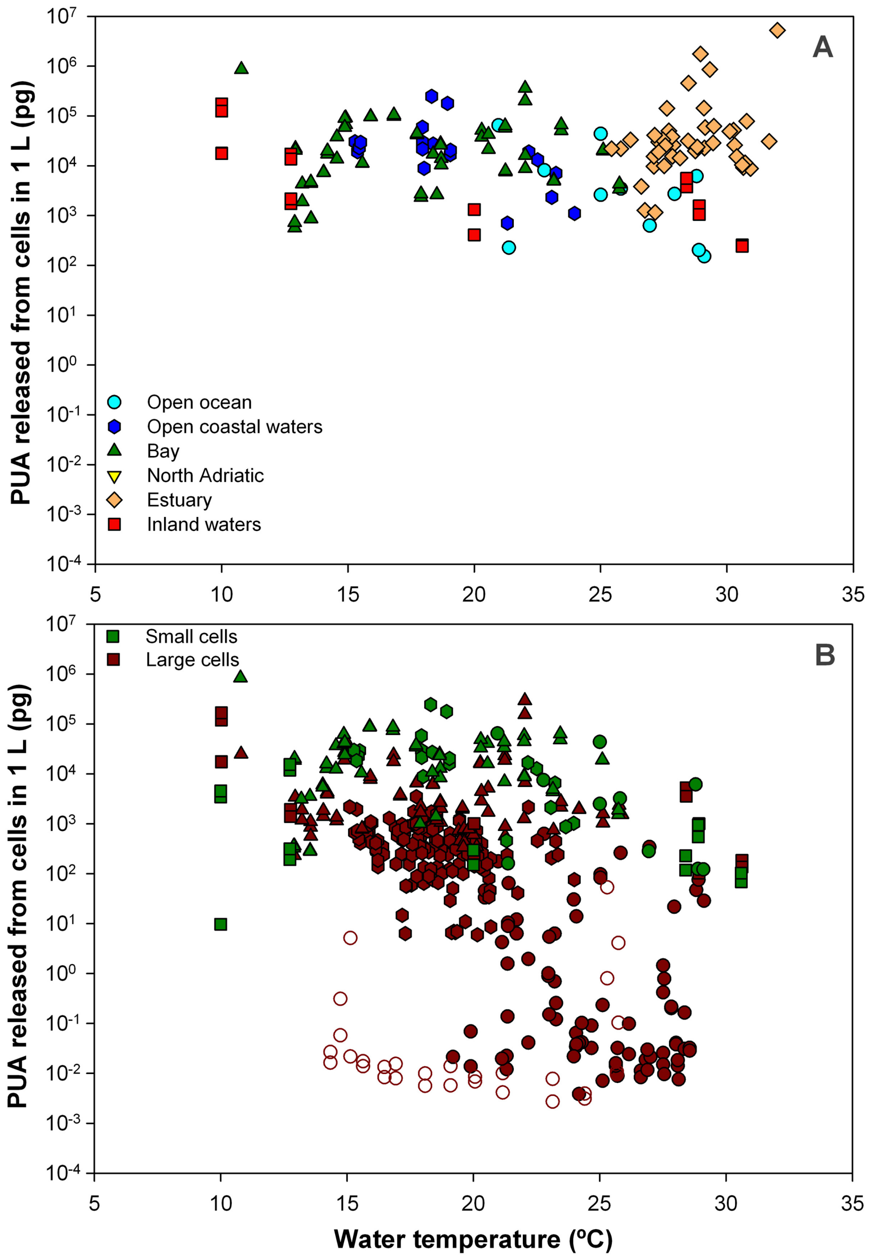

Figure S9: Total PUA released from the cells in 1-L sample (μg L^{-1}) in relation to water temperature. Whole phytoplankton assemblage (A), and small-size and large-size fractions (B). Empty symbols account for samples belonging to the Atlantic transect from Dominican Republic to NW Spain.

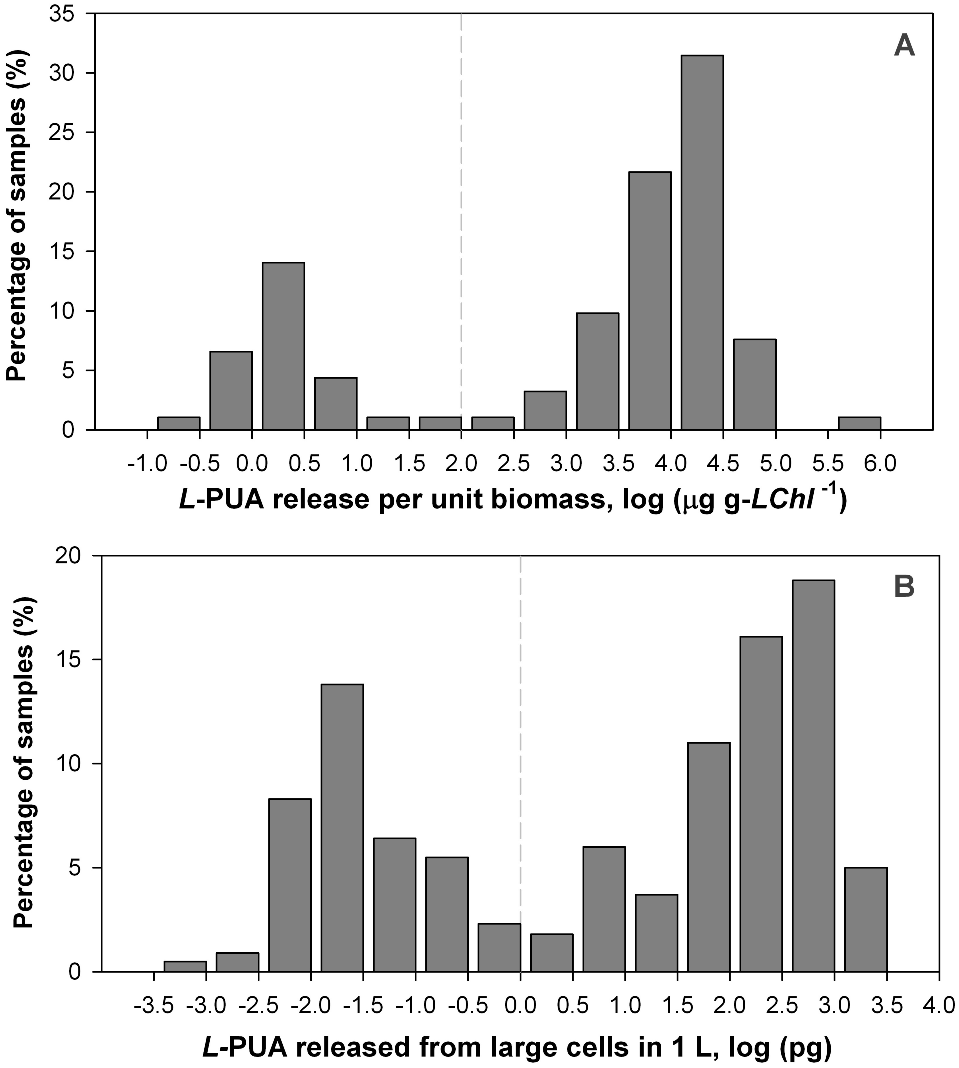

Figure S10: Frequency histogram of production of PUAs by large cells in oligotrophy (*TChl* < 1 μg L^{-1}) measured as PUA per unit biomass (A) and total PUAs (B). Data are graphed in figures 2B and 3B.
